# Cryptic diversity and recent diversification in the *Pyrrhulina australis* species complex (Characiformes, Lebiasinidae) revealed by mitochondrial DNA

**DOI:** 10.1111/jfb.70548

**Published:** 2026-07-02

**Authors:** Taina Barbosa De Souza, Daniela Cristina Ferreira, Hugmar Pains Da Silva, Carolina B. Machado, Claudio Oliveira, André L. Netto‐Ferreira, Paulo C. Venere

**Affiliations:** ^1^ Programa de Pós‐graduação em Ecologia e Conservação da Biodiversidade Universidade Federal de Mato Grosso Cuiabá Mato Grosso Brazil; ^2^ Laboratório de Citogenética e Genética Animal, Instituto de Biociências Universidade Federal de Mato Grosso Cuiabá Mato Grosso Brazil; ^3^ Faculdade de Ciências Agrárias, Biológicas, Engenharias e da Saúde Universidade Estadual de Mato grosso Tangará da Serra Mato Grosso Brazil; ^4^ Facultad de Ciencias Biológicas Universidad Nacional Mayor de San Marcos Lima Peru; ^5^ Departamento de Morfologia, Instituto de Biociências Universidade Estadual Paulista Júlio de Mesquita Filho Botucatu São Paulo Brazil; ^6^ Laboratório de Ictiologia, Departamento de Zoologia, Instituto de Biociências Universidade Federal do Rio Grande do Sul Porto Alegre Rio Grande do Sul Brazil

**Keywords:** Biogeography, COI gene, Lebiasinidae, Neotropical fishes, Pleistocene, Species delimitation

## Abstract

*Pyrrhulina australis* Eigenmann & Kennedy, 1903, is widely distributed across the main hydrographic basins of South America. The taxonomic integrity of this species is nevertheless challenged by the lack of clear diagnostic characters, its marked geographic variation and the morphological overlap with its congeners. In this context, the present study investigated the genetic diversity of the *P. australis* group (*P. australis*, *Pyrrhulina marilynae* and *Pyrrhulina vittata*), using sequences of the mitochondrial Cytochrome Oxidase I gene to delimit evolutionary lineages and provide insights into biogeographic patterns and divergence times. The molecular delimitation analyses (Automatic Barcode Gap Discovery [ABGD], Assemble Species by Automatic Partitioning [ASAP], Bayesian Poisson Tree Processes (bPTP), multi‐rate Poisson Tree Processes [mPTP], General Mixed Yule Coalescent [GMYC] and Poisson Tree Processes [PTP]) revealed 12 consensual MOTUs, which represent a complex of cryptic species revealed under the nominal name *P. australis*, with widely distributed lineages. Divergence time estimates indicate a recent evolutionary history for the group, occurring between the late Pliocene and the Pleistocene. The results reveal a surprising level of diversity within *P. australis* and provide unprecedented discussions on the biogeographic patterns of the group.

## 1 INTRODUCTION

*Pyrrhulina australis* Eigenmann & Kennedy, 1903, is a small South American fish of the family Lebiasinidae, which belongs to the order Characiformes. This species is found in the basins of the Amazon, Araguaia–Tocantins, Paraná/Paraguay and Uruguay rivers, as well as the local drainages of the South Atlantic coast (Bertaco et al., 2016; Dagosta & De Pinna, 2019; Toledo‐Piza et al., 2024; Venere & Garutti, 2011). Given its ample distribution, reliable identification of *P. australis* is a challenge, due not only to the considerable geographic variation found across this range (Zarske & Géry, 2004), but also the lack of clear diagnostic characters in the original description of the species and the reduced morphometric and meristic differentiation of the species from its congeners (Zarske & Géry, 2004). The overlap in morphological traits and the recurrent phylogenetic inconsistencies found among species further accentuate the challenges of delimiting *P. australi*s and its congeners.

The *P. australis* group was defined by Netto‐Ferreira & Marinho (2013) based on morphological characters and includes *P. australis*, *Pyrrhulina vittata* Regan, 1912, *Pyrrhulina marilynae* Netto‐Ferreira & Marinho 2013, *Pyrrhulina zigzag* Zarske & Géry, 1997, and *Pyrrhulina rachoviana* Myers, 1926. The status of *P. rachoviana* nevertheless remains unclear. This species was described based on two specimens obtained in 1906 from a locality considered by the author himself to be uncertain, given that the name—reported as Rosario—may refer to sites in Argentina, Paraguay or Uruguay, even though the subsequent literature has considered it to be in Argentina. Zarske and Géry (2004) present evidence that these specimens may in fact correspond to *P. australis*, whereas Zarske (2016) argued that these specimens may have originated from the Lower Amazon, specifically, Belém, Pará, Brazil and highlighted the presence of a primary black stripe, which brings their identity into doubt, although these conclusions were based on the author's deductions, rather than any specific empirical evidence. In that study, the author suggested that *P. marilynae* was in fact a junior synonym of *P. rachoviana*, due to its similarities in coloration, such as the presence of a longitudinal black stripe extending from the tip of the snout to the caudal peduncle (Zarske, 2016). In this context, the presence or absence of a primary black stripe along the body emerges as one of the principal sources of taxonomic uncertainty within the *P. australis* group.

In *P. australis*, the primary black stripe is restricted to the snout, whereas in other species of the group (i.e., *P. marilynae*, *P. rachoviana* and *P. zigzag*) the stripe extends along the flanks. Nevertheless, some specimens identified as *P*. aff. *australis* also exhibit a longitudinal black stripe, particularly those from populations of the Tietê and Upper Paraguay rivers and the Amazon basin (de Moraes et al., 2017, 2021; Netto‐Ferreira & Marinho, 2013; Zarske & Géry, 2004). In a cytogenetic study, de Moraes et al. (2017) recorded the same diploid number (2*n* = 40) in the two morphotypes, that is, *P. australis* (no stripe, Paraguay Basin) and *P*. aff. *australis* (stripe, Amazon Basin), although significant genomic differences were found. *Pyrrhulina* aff. *australis* differs from *P. australis* due to the presence of exclusively interstitial C‐banding, a reduced number and more restricted distribution of rDNA sites and more widely dispersed repetitive sequences, which together support their recognition as distinct evolutionary units (de Moraes et al., 2017).

De Moraes et al. (2021) subsequently included a larger number of striped populations, including *P*. aff. *australis* from the Sepotuba River (Paraguay Basin), *P*. aff. *marilynae* (Igarapé 12 de Outubro—Amazon Basin), *P. australis* (Paraguay Basin) and *P. marilynae* (Tapajós Basin). All the specimens presented 2*n* = 40, except *P. marilynae* (2*n* = 32). Despite the general conservation of the diploid number, the different morphotypes present genomic differences that reinforce their genetic diversification. As recent molecular analyses have revealed a high degree of genetic differentiation, that is, intraspecific distances of up to 3.74%, *P. australis* is currently considered to be a species complex. Souza et al. (2023) identified multiple monophyletic lineages, distributed in the Guaporé (Amazon), Araguaia and Upper Paraná basins, but recognized the populations from the Paraguay and Lower Paraná basins as a single evolutionary unit. These findings reflect a complex biogeographic history, given that the diversification of the ichthyofauna of South America is associated integrally with the geological history of the continent's principal hydrographic basins, in particular, the Orinoco–Amazon–Paraná–Paraguay system (Reis et al., 2016), which have been shaped by events such as the uplifting of the Andes, marine transgressions and the consolidation of the Brazilian and Guiana shields (Lundberg et al., 1998). These largely orogenic processes have defined the configuration of the lowland and highland zones of South America, which has, in turn, influenced its hydrographic connectivity and the associated evolutionary and biogeographic patterns of the region's ichthyofauna (Ribeiro et al., 2016).

In this context, the present study is based on the molecular identification of the *P. australis* populations found across the whole geographic distribution of the species and the delimitation of its Molecular Operational Taxonomic Units (MOTUs). The study also discusses the taxonomic limits and validity of the *P. australis* group (*P. australis, P. marilynae* and *P. vittata*), through the integration of the recent molecular evidence (Souza et al., 2023) with the hypotheses proposed by Netto‐Ferreira and Marinho (2013). Estimates of divergence times among the clades are also presented, providing insights for the assessment of the historical context of the diversification of the MOTUs and its biogeographic implications.

## 2 MATERIALS AND METHODS

### 2.1 Definition of the taxa and sampling

Specimens of *Pyrrhulina* were selected for molecular identification based on four criteria: (i) morphology; (ii) geographic distribution, with the aim of covering the greatest possible area of the known occurrence of *P. australis*, using the type locality—Arroyo Trementina in the Paraguay River basin at Concepción, Paraguay (22°47′00.9″ S, 56°52′59.1″ W; Fricke et al., 2026)—as a point of reference; (iii) the inclusion of all the lineages identified by Souza et al. (2023); and (iv) species related closely to *P. australis* in phylogenetic terms, based on the morphological criteria of Netto‐Ferreira and Marinho (2013), that is, *P. marilynae* and *P. vittata* (*P. rachoviana* and *P. zigzag* were not included due to the lack of genetic material for analyses). The COI barcode sequences of the *Pyrrhulina* specimens from Argentina were also added to the analyses. These specimens, obtained from the BOLD Systems as *P. australis*, include individuals collected from sites in the vicinity of the type locality of *P. rachoviana* in Rosario (accession numbers: LARI315‐13; LARI316‐13; MLPEC317‐12; MLPEC318‐12; MLPEC319‐12). Despite their geographic proximity to the type locality of *P. rachoviana*, all specimens were treated as *P. australis* in the present analyses.

**FIGURE 1:**
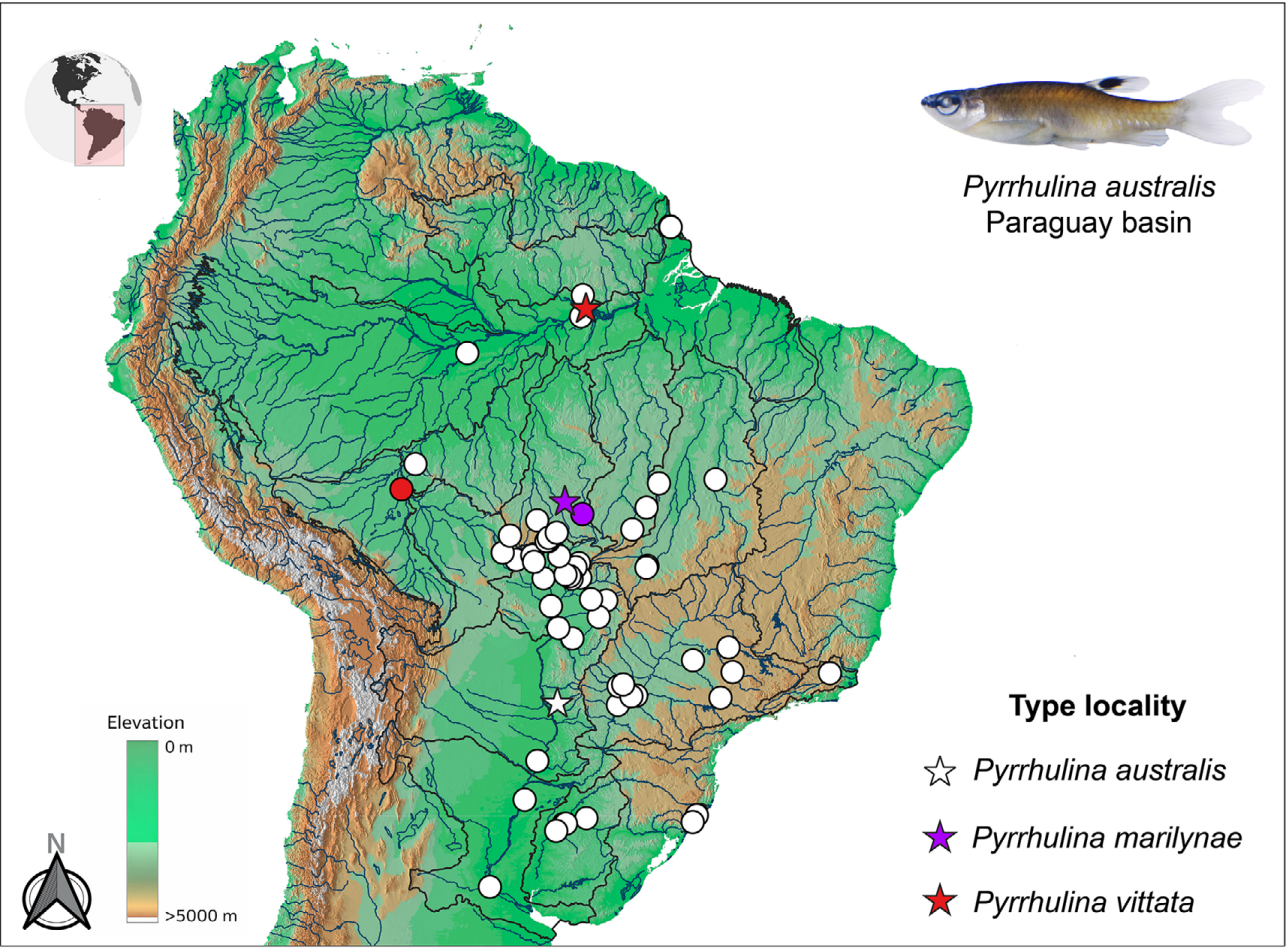
The collecting localities of the specimens analysed in the present study. The circles represent the sampling sites of the species of the *Pyrrhulina australis* group, which are colour‐coded by their morphological identification (see legend), while the type localities are represented by stars.

The geographic distribution of the specimens of the *P. australis* group analysed in the present study (Figure 1) encompasses the ecoregions of the Amazon Basin (Amazonian lowlands and coast—AML; Trombetas—TRO; Madeira—MAD; Guaporé—GUA; Purus—PUR; Mamoré—MAM; Tapajós—TAP and Xingu—XIN), the Tocantins–Araguaia basin (TOA), the Paraíba do Sul basin (PBS), the La Plata basin (Paraguay—PAR; Upper Paraná—UPA; Lower Paraná – LPA and Lower Uruguay—LUR) and the Tramandaí basin (TRA). *Pyrrhulina obermulleri* Myers, 1926, and *Pyrrhulina punctata* Netto‐Ferreira et al. (2026), both from the Amazonian lowlands of Peru, were included as outgroups.

Samples were obtained in the field during the present study (specimen collection authorized by SISBIO licence no. 15226–2) and also through partnerships with three Brazilian ichthyological collections, the Fish Biology Laboratory of São Paulo State University at Botucatu (Universidade Estadual Paulista: LBP–UNESP), the Ichthyology Laboratory at the Federal University of Rio Grande do Sul (UFRGS) in Porto Alegre and the Ichthyology Laboratory at the Federal University of Rondônia (LIP–UNIR), in Porto Velho. A total of 151 COI barcodes (*P. australis* group = 147; *Pyrrhulina* outgroup = 4) were used in the species delimitation analysis, which included 123 sequences generated in the present study and 28 *P. australis* sequences obtained from available public databases. For divergence time estimation among clades, an additional dataset comprising 46 outgroup COI barcodes was added to the dataset for the estimates of the divergence time among clades. These barcodes represent species of the families Bryconidae (*Brycon* Müller & Troschel, 1844; *Salminus* Agassiz, 1829; *Henochilus* Garman, 1890), Chalceidae (*Chalceus* Cuvier, 1818), Ctenoluciidae (*Boulengerella* Eigenmann, 1903; *Ctenolucius* Gill, 1861) and Lebiasinidae (*Lebiasina* Valenciennes, 1847; *Nannostomus* Günther, 1872; *Copella* Myers, 1956). The sequence accession numbers are provided in Tables S1 and S2.

### 2.2 Extraction and amplification of the DNA and sanger sequencing

The genomic DNA was extracted according to the protocol of Aljanabe and Martinez (1997). The Cytochrome Oxidase I gene (~650 base pairs) was amplified by Polymerase Chain Reaction (PCR) with the primers COI FISH F1 and FISH R1 (Ward et al., 2005). The final PCR volume of 25 μL contained 2.5 μL of buffer (10×), 1.5 μL of dNTPs (1.25 mM), 1.0 μL of MgCl_2_ (50 mM), 0.5 μL (10 mM) of each primer (Forward and Reverse), 0.1 μL of Taq DNA polymerase (Platinum, 5 U/μL), 1 μL of the DNA (~30 ng/μL) and ultrapure autoclaved water to complete the final volume. The thermal cycling conditions consisted of initial denaturation at 95°C (5 min), followed by 30 cycles of denaturation at 95°C (45 s), annealing at 52°C (30 s) and extension at 72°C (45 s), with a final extension of 7 min at 72°C.

The samples were purified and sequenced by a privately owned company, Biotecnologia Pesquisa e Inovação (BPI, Botucatu, São Paulo, Brazil; https://bpibiotecnologia.com.br). The amplicons were purified using an AMPure XP magnetic bead kit and sequenced using a BigDye® Terminator v.3.1 Cycle Sequencing kit (Applied Biosystems). The ethanol/EDTA/sodium acetate precipitation reaction was based on the protocol suggested by the manufacturer for the use of AMpure XP magnetic beads. All the sequences generated in the present study were deposited in GenBank, under the accession numbers provided in Table S1.

### 2.3 Data editing, sequence alignment and analysis

The raw sequences were edited in Geneious® 7.1.3 (Kearse et al., 2012) and inspected for gaps and stop codons. The final consensus sequences were constructed and aligned using the Muscle algorithm (Edgar, 2004) in the MEGA v.11.0 programme (Tamura et al., 2021). This same programme was used to identify pseudogenes, deletions and insertions. The saturation of nucleotide substitutions was evaluated in DAMBE7 (Xia et al., 2003).

The MOTUs were delimited based on the Automatic Barcode Gap Discovery (ABGD; Puillandre et al., 2012) and Assemble Species by Automatic Partitioning (ASAP; Puillandre et al., 2021) analytical procedures, which are based on genetic distances. These procedures were complemented by four coalescence‐based methods—the General Mixed Yule Coalescent (GMYC; Pons et al., 2006), multi‐rate Poisson Tree Processes (mPTP; Kapli et al., 2017), Poisson Tree Processes (PTP; Zhang et al., 2013) and the Bayesian implementation of the bPTP (Zhang et al., 2013). All these procedures were implemented in the SPDEL pipeline (Ramirez et al., 2023), which was run with default parameters in the Anaconda v.25.1.1 environment within the Ubuntu v.24.04.1 terminal. This pipeline generates individual and consensual estimates of the MOTUs, as well as calculating means genetic distances and the maximum intra and minimum inter‐MOTU distances between lineages, based on the K2P model (Kimura, 1980).

An alignment in the FASTA format was used as the input for the ABGD and ASAP procedures. In the case of the coalescence‐based methods (mPTP, PTP and GMYC), an ultrametric tree, compiled in BEAST2 v.2.6.7 (version available on CIPRES; https://www.phylo.org/), was also used as input, in addition to the FASTA alignment. The tree was generated with the HKY evolutionary model, which was selected using jModelTest2 (Darriba et al., 2012), with a relaxed molecular clock and a Birth–Death tree prior, using three independent Markov Chain Monte Carlo (MCMC) runs of 50 million generations. Effective sample sizes (ESS > 200) were assessed subsequently in Tracer v.1.6. The trees were combined in LogCombiner v.2.6.6 with a 25% burn‐in, summarized in TreeAnnotator v.2.6.6, visualized in FigTree v.1.4.3 and then saved in ‘.nwk’ (Newick) format.

The maximum intraspecific and minimum interspecific genetic distances among the consensus MOTUs generated by the SPDel pipeline were plotted on a graph in Excel. The quadrants were classified following Hebert et al. (2004), although the Optimal Threshold (OT) was calculated specifically for the dataset in the R environment using the LocalMinima function of the SPIDER package (SPecies IDentify and Evolution in R; Brown et al., 2012). The quadrants were defined as follows: (I) total agreement between the morphological and molecular identification; (II) potentially cryptic species; (III) possible recent divergence, hybridization or synonymy; and (IV) discrepancy between the morphological and molecular identifications. A haplotype network was constructed using the median‐joining algorithm in PopArt v.1.7 (Leigh & Bryant, 2015) to provide a visual representation of the genetic structure and diversity of the MOTUs of the *P. australis* group. The species identified here were treated as MOTUs for the interpretation of the evolutionary units, with the term ‘genetic lineage’ being applied to the individuals identified morphologically as *P. australis*, in accordance with the unified species concept, which considers species to be distinct lineages of a metapopulation that have evolved independently (De Queiroz, 2007).

A coalescent tree was generated in BEAST2 to estimate the divergence times among the MOTUs of the *P. australis* group, based on the following parameters: (i) Birth–Death speciation model, (ii) relaxed molecular clock and (iii) a mutation rate of 0.01 per million years for the COI gene, based on the estimate of Bermingham et al. (1997) for fish mitochondrial genes, calibrated using the closest known fossil of the Lebiasinidae, †*Brycon avus* (Woodward, 1898), dated to the Oligocene, approximately 23.8 million years ago (Ma). The full dataset comprised 197 COI barcodes of species (ingroup = 151; outgroup = 46; Table S2) of the families Bryconidae (*Brycon*; *Salminus*; *Henochilus*), Chalceidae (*Chalceus*), Ctenoluciidae (*Boulengerella*; *Ctenolucius*) and Lebiasinidae (*Lebiasina*; *Nannostomus*; *Copella*). The tree was compiled using the same parameters as those adopted by Melo et al. (2022), except for the exact node calibration. A number of different tests were run, and the divergence time estimate most consistent with the fossil record was obtained by calibrating the Most Recent Common Ancestor (MRCA) of all the bryconid species (Figure S1). These analyses were based on three independent runs of 110 million generations, which were sampled every 110 thousand generations.

## 3 RESULTS

### 3.1 Molecular delimitation of species

The final alignment of the 151 COI sequences of *Pyrrhulina* resulted in a total of 620 base pairs (bp), of which 490 were conserved and 112 were parsimony‐informative for the analyses. No insertions, deletions or stop codons were identified in this dataset. The nucleotide substitution saturation index (ISS) indicated a low level of saturation in the dataset, given that the observed value (*R* ^2^ = 0.082) was considerably lower than the critical ISS (*R* ^2^ = 0.740), which reinforces the reliability of the data for the analyses.

**FIGURE 2:**
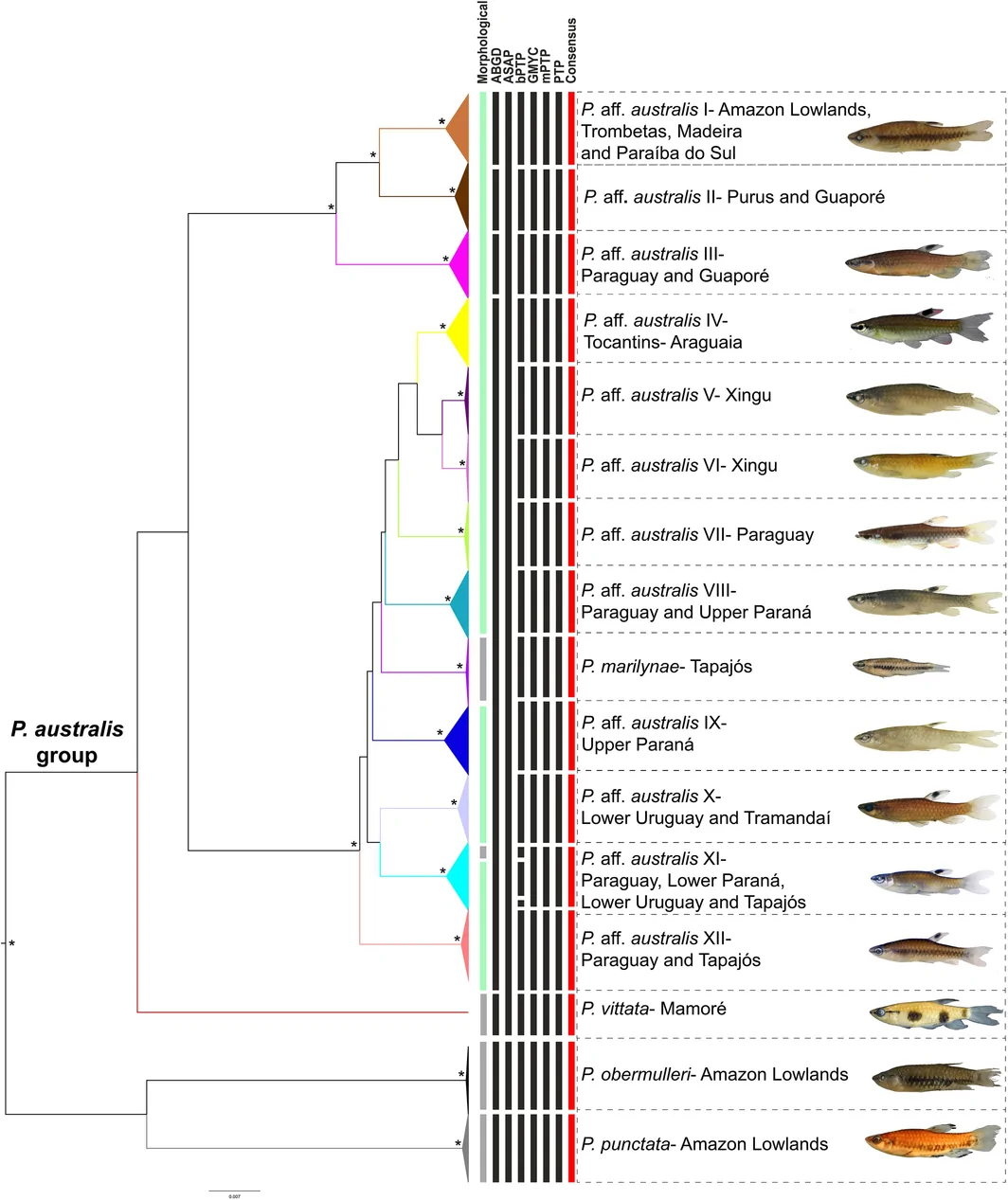
Plot of the results of the Bayesian inference analysis of the Cytochrome Oxidase I sequence data run in BEAST2. The asterisks indicate a posterior probability of more than 95%. The vertical bars at the left indicate the a priori morphological identification of each lineage as either *Pyrrhulina australis* (green) or a different *Pyrrhulina* species (grey), while the black bars in the middle columns delimit the Molecular Operational Taxonomic Units (MOTUs) recognized in the species delimitation analyses. The red bars at the left indicate the consensus MOTUs, with each clade being indicated by a distinct colour (triangles at left), corresponding to the MOTUs. The images of *P*. aff. *australis* IV, *P. vittata* and *P. punctata* were adapted from Venere and Garutti (2011), Marinho and Netto‐Ferreira (2014) and Netto‐Ferreira et al. (2026), respectively.

The SPDel pipeline recognized 16 consensual MOTUs, based on a combination of the different molecular identification methods applied here (Figure 2). Twelve of these lineages had been identified previously as *P. australis*: *P*. aff. *australis* I (Amazonian lowlands and coast—AML; Trombetas—TRO; Madeira—MAD and Paraíba do Sul—PBS), *P*. aff. *australis* II (Purus—PUR and Guaporé—GUA), *P*. aff. *australis* III (Paraguay—PAR and GUA), *P*. aff. *australis* IV (Tocantins–Araguaia—TOA), *P*. aff. *australis* V (Xingu—XIN), *P*. aff. *australis* VI (XIN), *P*. aff. *australis* VII (PAR), *P*. aff. *australis* VIII (PAR and Upper Paraná—UPA), *P*. aff. *australis* IX (UPA), *P*. aff. *australis* X (Lower Uruguay—LUR and Tramandaí—TRA), *P*. aff. *australis* XI (PAR; Lower Paraná—LPA; LUR and Tapajós—TAP) and *P*. aff. *australis* XII (PAR and TAP). The MOTUs of *P. marilynae* (TAP), *P. vittata* (Mamoré—MAM), the outgroups *P. obermulleri* (AML) and *P. punctata* (AML) formed distinct clades, all with high statistical support (Figure 2). These results corroborate the hypothesis that the *P. australis* group consists of a single, monophyletic group (*P. vittata* [*P*. aff. *australis*, *P. marilynae*]).

The coalescence‐based methods (GMYC, bPTP, mPTP and PTP) recovered 12 distinct lineages from the *P. australis* sequences. By contrast, the genetic distance–based methods (ASAP and ABGD) provided a less clear resolution. In fact, ABGD identified only four *P. australis* lineages, with *P. marilynae* being assigned to one of these MOTUs, while *P. vittata* was kept separate, and ASAP grouped *P. australis*, *P. marilynae* and *P. vittata* in a single MOTU (Figure 2).

The MOTUs identified here returned mean intra‐MOTU values well below the Optimal Threshold (OT = 1.01%), close to zero, in fact (Table 1). However, the minimum inter‐MOTU genetic distances were also relatively low, in comparison with the OT, with a lowest value of 0.49% among *P*. aff. *australis* lineages V (XIN), VI (XIN) and VII (PAR and UPA). By contrast, the maximum intra‐MOTU distances were very close to the OT, in particular in *P*. aff. *australis* I lineages (AML, TRO, MAD and PBS), IV (TOA) and IX (UPA), which returned intraspecific divergence values of 0.99%. These findings may account for the lower resolution obtained in the genetic distance–based methods (ASAP and ABGD).

**TABLE 1:** Mean and maximum intra‐MOTU genetic distances and the minimum inter‐MOTU distance (%) to the Nearest Neighbor (NN) recorded in the *Pyrrhulina* lineages identified in the present study.

*Pyrrhulina* MOTUs	Mean intraespecific	Max intraespecific	Nearest neighbour (NN)	Distance to NN
*P*. aff. *australis* I (Amazon lowlands and coastal region, Trombetas, Madeira and Paraíba do Sul)	0.37	0.99	*P*. aff. *australis* II (Purus and Guaporé)	2.34
*P*. aff. *australis* II (Purus and Guaporé)	0.49	0.49	*P*. aff. *australis* I (Amazon lowlands and coastal region, Trombetas, Madeira and Paraíba do Sul)	2.34
*P*. aff. *australis* III (Paraguay and Guaporé)	0.16	0.49	*P*. aff. *australis* I (Amazon lowlands and coastal region, Trombetas, Madeira and Paraíba do Sul)	2.85
*P*. aff. *australis* IV (Tocantins‐ Araguaia)	0.48	0.99	*P*. aff. *australis* V (Xingu)	0.99
*P*. aff. *australis* V (Xingu)	0	0	*P*. aff. *australis* VI (Xingu) and P. aff. *australis* VII (Paraguay and Upper Paraná)	0.49
*P*. aff. *australis* VI (Xingu)	0	0	*P*. aff. *australis* V (Xingu)	0.49
*P*. aff. *australis* VII (Paraguay)	0	0	*P*. aff. *australis* V (Xingu)	0.65
*P*. aff. *australis* VIII (Paraguay and Upper Paraná)	0.19	0.58	*P*. aff. *australis* V (Xingu)	0.49
*P*. aff. *australis* IX (Upper Paraná)	0.32	0.99	*P*. aff. *australis* VII (Paraguay and Upper Paraná)	1.15
*P*. aff. *australis* X (Uruguay and Tramandaí)	0.1	0.32	*P*. aff. *australis* VII (Paraguay and Upper Paraná)	1.32
*P*. aff. *australis* XI (Paraguay, Lower Paraná, Lower Uruguay and Tapajós)	0.2	0.84	*P*. aff. *australis* VII (Paraguay and Upper Paraná)	1.32
*P*. aff. *australis* XII (Paraguay and Tapajós)	0	0	*P*. aff. *australis* VII (Paraguay and Upper Paraná)	1.76
*P. marilynae* (Tapajós)	0	0	*P*. aff. *australis* VII (Paraguay and Upper Paraná)	0.82
*P. vittata* (Mamoré)	‐	‐	*P*. aff. *australis* VII (Paraguay)	5.49
*P. obermulleri* (Amazon lowlands)	0	0	*P. punctata* (Amazon lowlands)	7.32
*P. punctata* (Amazon lowlands)	0.17	0.17	*P*. aff. *australis* VII (Paraguay and Upper Paraná)	7.26

**FIGURE 3:**
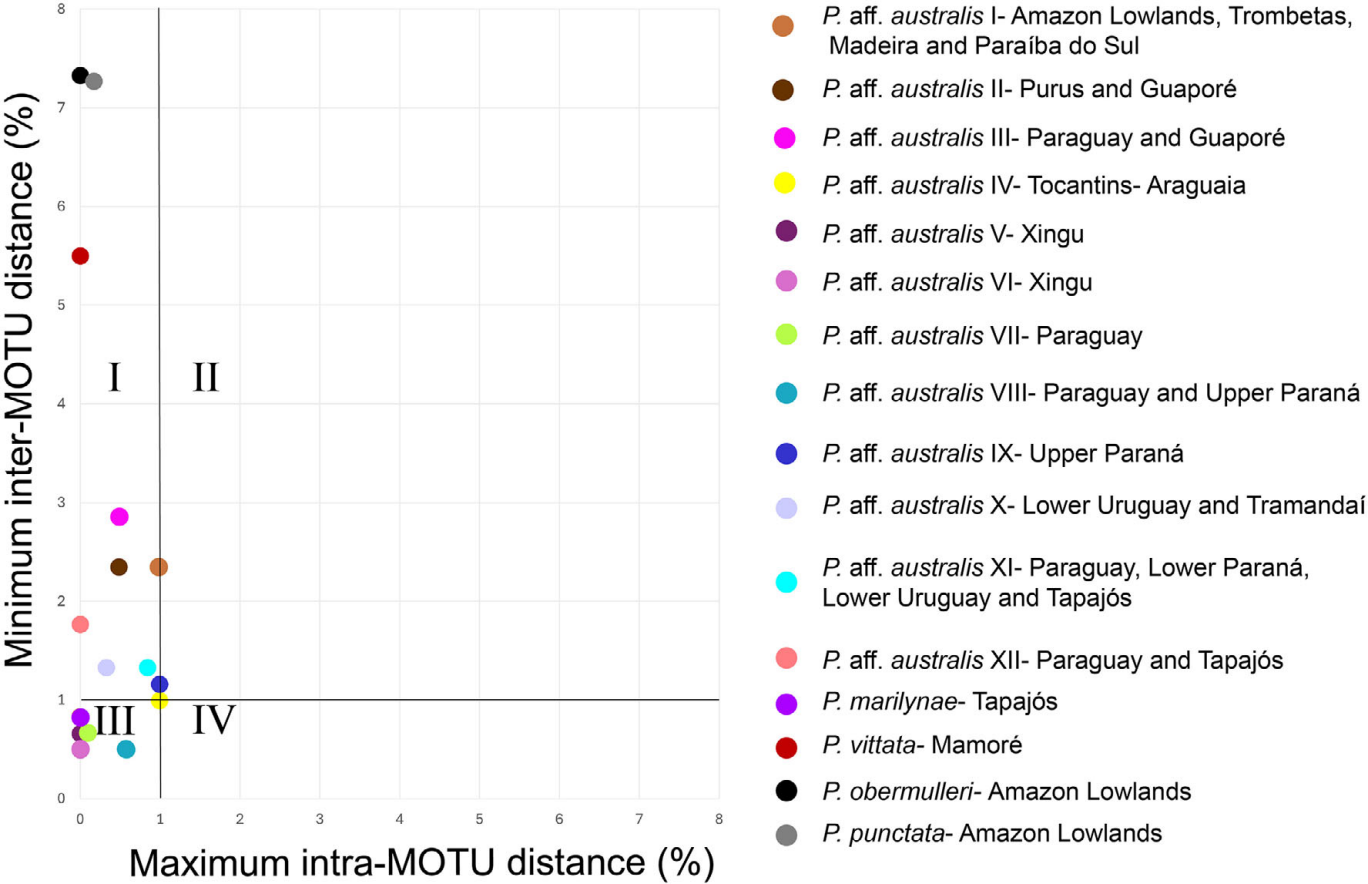
Quadrant plot: The solid lines represent an OT = 1%, separating the values (%) on the axes corresponding to the minimum inter‐MOTU and maximum intra‐MOTU distances. The quadrants correspond to: (I) congruent morphological and molecular identifications; (II) potential cryptic species; (III) possible recent divergence, hybridization or synonymy; and (IV) morphology not corresponding to the molecular identification. The circles represent the consensus MOTUs of *Pyrrhulina* aff. *australis* lineages I–XII, *P. marilynae*, *P. vittata*, *P. obermulleri* and *P. punctata*.

Most of the MOTUs were plotted in quadrant I (Figure 3), which means that their molecular classification corresponded exactly to their morphological identification. However, the morphological identification was not used as a criterion for this division, as the groupings were based on consensual MOTUs. Despite being based on different algorithms, the distinct methods yielded consistent results. This congruence was observed even where the genetic distances were very low, such as in the lineages of *P*. aff. *australis* (V—XIN; VI—XIN; VII—PAR; VIII—PAR and UPA) and *P. marilynae* (TAP), which were assigned to quadrant III (Figure 3), which indicates possible recent divergence, hybridization or synonymy.

### 3.2 Geographic distribution of the *P. australis* lineages

**FIGURE 4:**
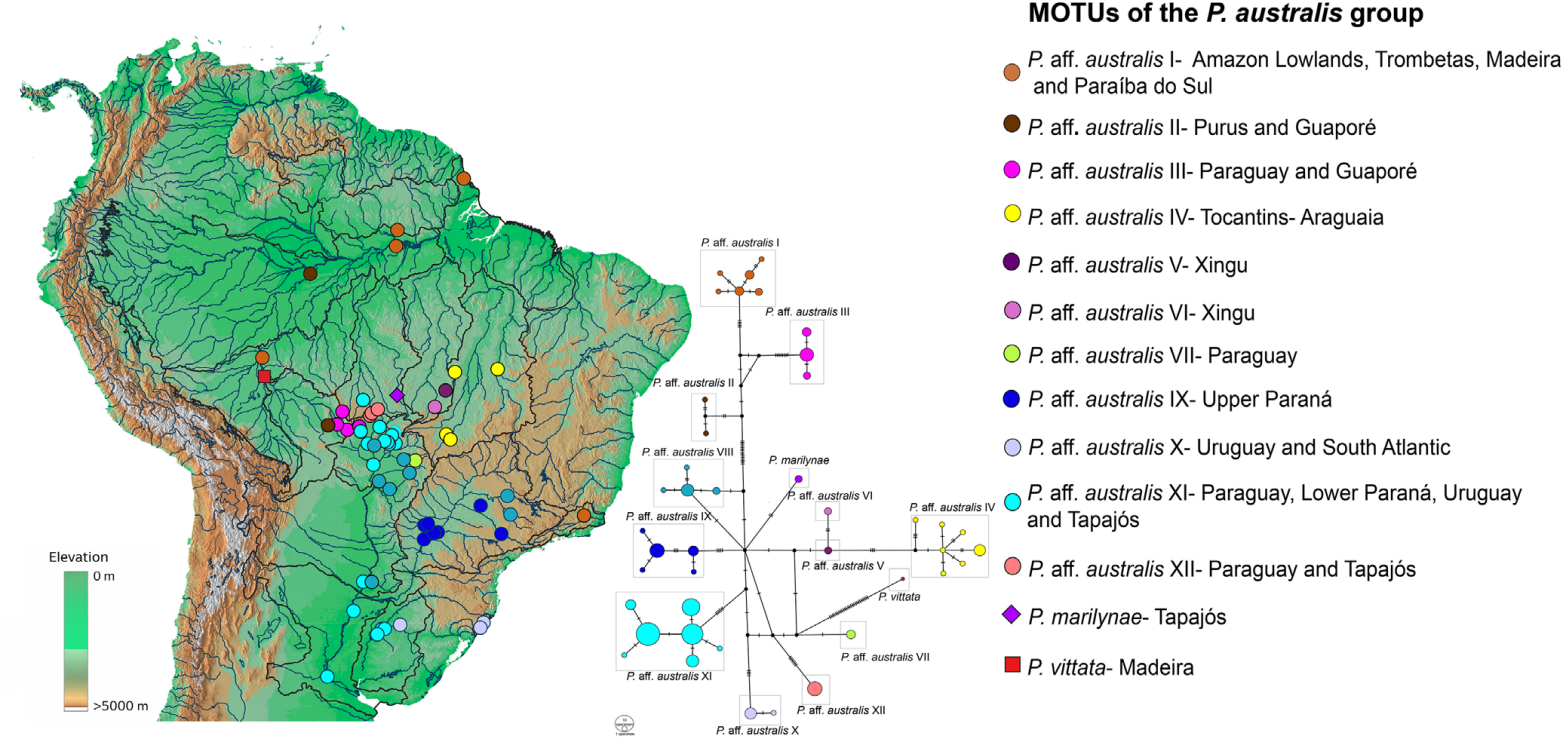
Haplotype network of the COI sequences of the *Pyrrhulina australis* group recorded in the present study, constructed in PopArt. Each circle represents a single haplotype, with its size proportional to its frequency and colour‐coded according to the MOTU (see legend). The small black dot represents the missing haplotypes. The collecting points plotted on the map are also colour‐coded by their consensus MOTU.

The structure of the haplotype network revealed a high level of genetic diversity and a close relationship among the *P*. aff. *australis* lineages, *P. marilynae* and *P. vittata* (Figure 4). Overall, the MOTUs encompassed 42 haplotypes, with none of the haplotypes being shared between lineages. *P. vittata* has the largest number of mutational steps in comparison with the other lineages, whereas some *P*. aff. *australis* MOTUs (i.e., lineages V, VI and VII) are separated by only a few mutational steps, which reflects the reduced genetic distances recorded here (Table 1) and justifies their presence in quadrant III (Figure 3).

The distribution of the MOTUs of the *P. australis* group among the different hydrographic basins of South America, as well as the inclusion of specimens from different localities both within the same basin and from different basins, revealed a considerable degree of intraspecific genetic structuring within each MOTU. Individuals of *P*. aff. *australis* lineage I (AML, TRO, MAD and PBS) had low intra‐MOTU genetic divergence (0.37%) and some individuals from the Amazon and Paraíba do Sul rivers share a haplotype, despite the geographic disjunction of these hydrographic basins. Similarly, *P*. aff. *australis* XI share seven haplotypes separated by only a few mutational steps but are widely distributed across the upper and middle Paraguay, lower Paraná and Uruguay and Tapajós basins (Figure 4).

The geographic distribution of the *P. australis* lineages does not align strictly with hydrographic boundaries (Figure 4). In fact, while five of the 12 identified lineages are restricted to a single basin (i.e., lineages IV—TOA, V and VI—XIN, VII—PAR and IX—UPA), they are sympatric with other *P*. aff. *australis* lineages in these basins. The other seven lineages are found in more than one basin (I—AML, TRO, MAD and PBS; II—PUR and GUA; III—PAR and GUA; VIII—PAR and UPA; X—LUR and TRA; XI—PAR, LPA, LUR and TAP; XII—PAR and TAP).

Syntopy between lineages was also identified in two cases. Specimens of *P*. aff. *australis* lineages II and III were collected at Vila Bela da Santissima Trindade, in the Guaporé basin of Mato Grosso, while individuals representing lineages VIII and XI were captured from both the Bento Gomes River in the Paraguay Basin, in Poconé (Mato Grosso), Brazil and Formosa, in the Lower Paraná Basin, in Argentina.

### 3.3 Diversification of the *P. australis* group

**FIGURE 5:**
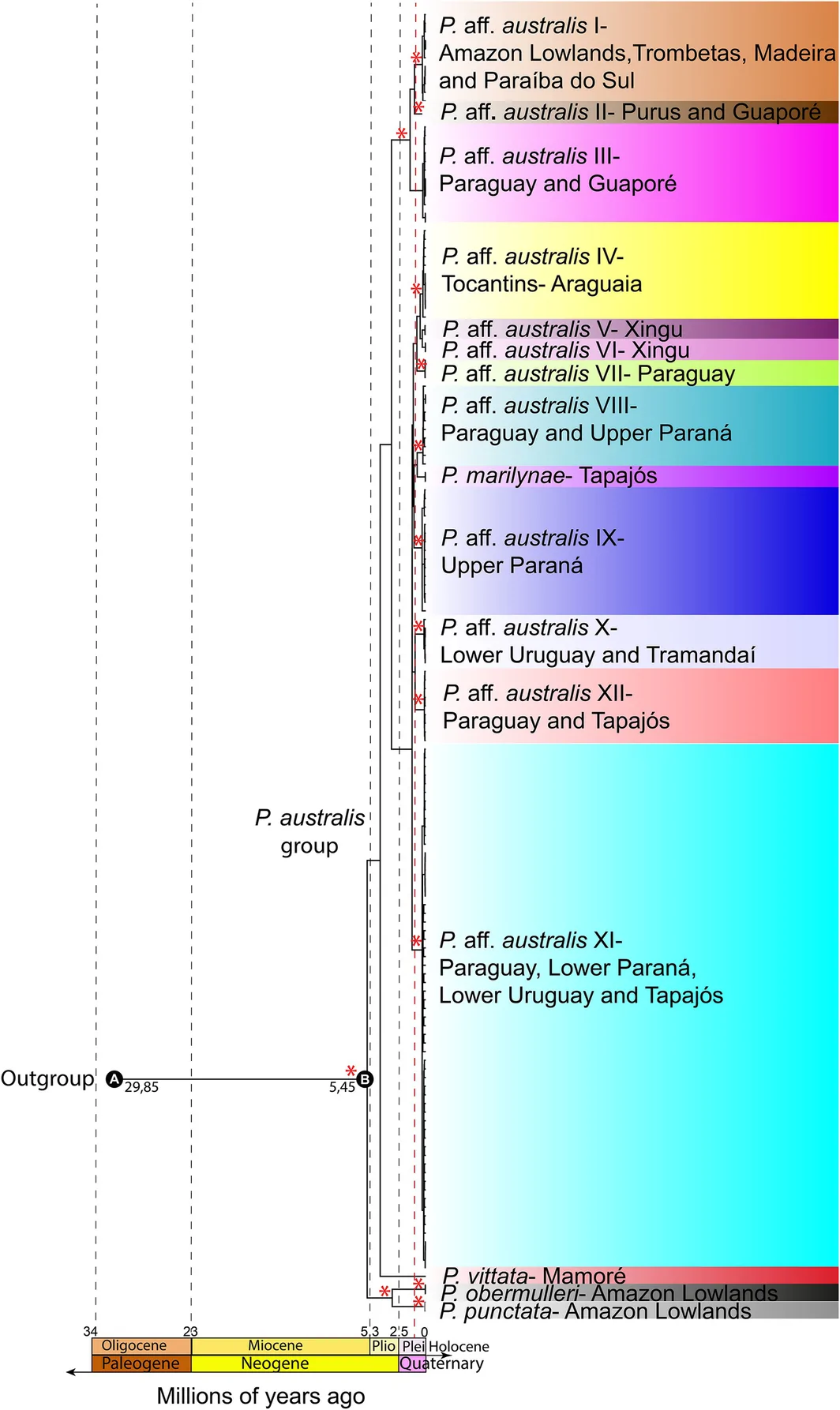
Time‐calibrated Bayesian tree for the MOTUs of the *Pyrrhulina australis* group based on the estimated mutation rate of the COI gene and the age of the fossil †*Brycon avus*. Divergence time estimates (in millions of years ago, Ma) are shown on the horizontal axis, with the geological periods being delimited by the vertical black dashed lines. The red dashed line indicates the timeline of the most recent lineages during the Pleistocene. Point A indicates the divergence between the Most Recent Common Ancestor (MRCA) of *Pyrrhulina* and *Copella* (Oligocene), and point B, the MRCA of the *P. australis* group and its Amazonian congeners (*P. obermulleri* and *P. punctata*). Red asterisks on branches indicate posterior probabilities of more than 95%. The consensus MOTUs are colour‐coded according to their geographic distribution.

Divergence time estimates indicate that the split between the MRCA of *Pyrrhulina* and *Copella* occurred during the Oligocene, approximately 29.85 Ma (Figure 5; 95% Highest Posterior Density [HPD] = 11.48–53.02 Ma). The MOTUs of the *P. australis* group are relatively recent, having diverged from Amazonian congeners (*P. obermulleri* and *P. punctata*) in the late Miocene, around 5.45 Ma (95% HPD = 2.39–8.98 Ma; Figure 5). The diversification of *P. vittata* from the remaining MOTUs of the *P. australis* group occurred during the Pliocene, at approximately 4.24 Ma (95% HPD = 1.74–7.33 Ma), followed by a second Pliocene event at around 3.19 Ma (95% HPD = 1.49–5.44 Ma), which gave rise to the clade composed of *P*. aff. *australis* lineages I (AML, TRO, MAD and PBS), II (PUR and GUA) and III (PAR and GUA). The remaining lineages of the *P. australis* group (including *P. marilynae*) diversified more recently, during the Pleistocene. The divergence times found among the outgroups are provided in Figure S1.

## 4 DISCUSSION

### 4.1 Molecular delimitation of species

The results presented here reveal considerable genetic diversity within *P. australis*, with a total of 12 evolutionary lineages identified consensually by the six different species delimitation methods (ABGD, ASAP, GMYC, PTP, bPTP and mPTP) applied in the analyses. This broad‐scale cryptic diversity has been discussed in several previous studies that have discussed the high levels of hidden diversity found across the ample distribution of the species (Souza et al., 2023; Netto‐Ferreira & Marinho, 2013; Zarske & Géry, 2004). In this context, it becomes increasingly necessary to integrate multiple approaches based on both genetic distance and coalescence‐based approaches and to evaluate their consistency systematically to better ensure the accuracy of the delimitation of lineages (Carstens et al., 2013). The combined application of these different approaches has proven especially relevant for the analysis of species complexes and cryptic groups of Neotropical fishes (Arruda et al., 2019; Fernandes & Martins‐Santos, 2002; Guimarães et al., 2022; Oliveira‐Silva et al., 2024). While classical taxonomy remains indispensable, complementary investigation of both morphological traits and molecular evidence is necessary to refine the separation of taxa and improve their formal recognition.

Relatively large genetic distances, when associated with morphological differences and geographic dispersal, typically reflect an accumulation of mutations that is consistent with the presence of species boundaries, supporting the inference of species‐level differentiation (Bickford et al., 2007). However, this level of divergence is not always evident, particularly in recently evolved lineages, as observed here in the *P. australis* lineages. In this case, the estimated OT was low (1.01%), and some lineage boundaries were well below this threshold (0.49%, Table 1), which places them in quadrant III (Figure 3), a classification indicating recent divergence, hybridization or even possible synonymy. While a 2% threshold is often applied as the standard for fishes (Pereira et al., 2013; Ward, 2009), several studies have shown that reduced genetic distances hamper the identification of species complexes assigned to the ‘grey zone’ (De Queiroz, 2007). Examples of this scenario include *Characidium bimaculatum* and *Characidium deludens* (genetic distance = 0.9%; Oliveira‐Silva et al., 2024), *Leporinus* cf. *friderici* and *Leporinus piau* (0.4%; dos Reis et al., 2025) and some *Hypostomus* species that are morphologically distinct but have interspecific distances of 0.1% (Anjos et al., 2020). These results reinforce the conclusion that, while thresholds of this type may provide a reference point, they should not be applied in isolation, but rather, within an integrated framework that combines multiple lines of evidence for more reliable species delimitation.

The presence of the primary black stripe on the flanks, which has often been used as a diagnostic trait in studies of *Pyrrhulina* (Netto‐Ferreira, 2010), varied considerably among phylogenetically closely related species. For example, the stripe is absent from *P. australis* sensu stricto and present in *P. marilynae* and *P. zigzag*, while also being found in some populations identifiable as *P. australis* (Tietê and Upper Paraguay basins), as well as being relevant to the sexual dimorphism of the morphotype from the Lower Amazon designated as *P. rachoviana* (Netto‐Ferreira & Marinho, 2013; Zarske, 2016; Zarske & Géry, 2004). The results of the present study, nevertheless, indicate that the presence or absence of this stripe does not constitute an exclusive character of a single species, given that the lineages that present this trait—*P*. aff. *australis* I (AML, TRO, MAD and PBS), *P*. aff. *australis* XII (PAR and TAP) and *P. marilynae* (TAP)—do not form a single monophyletic group. In the specific case of *P*. aff. *australis* I, for example, the stripe was present or absent in individuals from the same locality (Figure S2), indicating intra‐population variation. These findings indicate that the black stripe found in the *P. australis* lineages is not a reliable diagnostic character when considered on its own and may simply reflect phenotypic plasticity. Similar variation in coloration patterns has already been observed within a single species of *Bryconops* and have been attributed to environmental factors such as pH, temperature and current velocity (Gonçalves et al., 2024; Sidlauskas et al., 2006).

Given the controversy surrounding the type locality of *P. rachoviana*, originally assigned to Rosario (Argentina) but later considered by Zarske (2016) to belong to the Lower Amazon (Belém, Pará, Brazil), specimens from both regions were included in the analyses (Table S1). The analysed individuals belong to the *P. australis* group but were recovered in distinct lineages: Amazonian specimens were assigned to *P*. aff. *australis* I, whereas specimens from Argentina were included in *P*. aff. *australis* XI, together with populations from the Paraguay, Tapajós and Uruguay basins. In contrast, *P. marilynae*, considered by Zarske (2016) as a junior synonym of *P. rachoviana*, was consistently recovered by the delimitation methods as a distinct MOTU from the other lineages mentioned above. These results support its validity as a species, as previously indicated by cytogenetic and molecular evidence reported in the literature (de Moraes et al., 2021, 2023; Ferreira et al., 2022; Souza et al., 2023). However, the absence of comparative morphological analyses involving the type material of *P. rachoviana* prevents more precise taxonomic conclusions, leaving the taxonomic status of this species uncertain and reinforcing the need for an integrative taxonomic revision of the group.

The Bayesian tree indicates that the species of the *P. australis* group form a clade (*P. vittata* [*P*. aff. *australis*, *P. marilynae*]). This is consistent with the morphological evidence discussed by Netto‐Ferreira and Marinho (2013) and is supported by diagnostic characters such as the reduction in the number of maxillary teeth. Within the *P. australis* clade, *P. vittata* and *P. marilynae* also exhibit a reduction in certain skeletal structures, including a reduced number of precaudal vertebrae and dorsal‐fin rays, as well as non‐elongated anal and caudal fins in the males. The variation in the structure of the nasal foramen and in the presence or reduction of postcleithrum 2 further reinforce this phylogenetic relationship. As this inference is based on a single locus, however, it would be necessary to increase the number of genetic markers to confirm these relationships more definitively through the compilation of a more reliable species tree. Even so, the position of *P. marilynae* within the clade containing the *P. australis* lineages confirms the close evolutionary relationship of these forms and corroborates previous genomic analyses that have recognized them as sister species (Ferreira et al., 2022).

The present study revealed that the material identified nominally as *P. australis* obscures a high level of diversity associated with the ample geographic distribution of the 12 lineages found here—*P*. aff. *australis* (lineages I‐XII), *P*. aff. *australis* I (AML, TRO, MAD and PBS), *P*. aff. *australis* II (PUR and GUA), *P*. aff. *australis* III (PAR and GUA), *P*. aff. *australis* IV (TOA), *P*. aff. *australis* V (XIN), *P*. aff. *australis* VI (XIN), *P*. aff. *australis* VII (PAR), *P*. aff. *australis* VIII (PAR and UPA), *P*. aff. *australis* IX (UPA), *P*. aff. *australis* X (LUR and TRA), *P*. aff. *australis* XI (PAR, LPA, LUR and TAP) and *P*. aff. *australis* XII (PAR and TAP). A new sequence of *P. marilynae* (TAP) is also presented, together with novel data on *P. obermulleri* (AML) and *P. vittata* (MAM). These findings indicate that the scientific understanding of the diversity of the group is still limited, which highlights the urgent need for a taxonomic review of the genus *Pyrrhulina*, which has not been revised since Regan (1912). The data also reinforce the importance of further efforts toward the description of the potential new species identified here, in addition to the assessment of their conservation status, given that *P. australis* does not represent a single widely distributed species, but rather, multiple evolutionary lineages that require a more detailed analysis.

### 4.2 Geographic distribution of the *P. australis* lineages

Watersheds act as natural barriers that limit the dispersal of aquatic organisms and promote endemism, as observed in the Amazon Basin, where approximately 63% of the fish species are endemic (Dagosta & De Pinna, 2019). This physical isolation is not the only factor explaining the diversification of species, however, considering that the geographic ranges of many taxa extend between different basins (see Lima & Ribeiro, 2011; Ribeiro et al., 2016). In the specific case of *Pyrrhulina australis*, the hypothesis of isolation by hydrographic watersheds, proposed by Souza et al. (2023) for the six evolutionary lineages identified in this study, may have been, at least in part, a result of sampling limitations, which may have masked broader distributional patterns. In the present study, the expansion of the geographic distribution of *P. australis*, albeit not as a single species, indicates that the lineages identified here encompass a paraphyletic group whose distribution exceeds hydrographic boundaries, reflecting certain, well‐known biogeographic patterns found in some Neotropical fishes.

*Pyrrhulina* aff. *australis* I lineage has a unique distribution, occurring in two distinct basins that are currently isolated geographically—the lower Amazon basin (including the Trombetas and Madeira rivers and the coastal region) and the coastal Paraíba do Sul basin, within the area of the Brazilian Shield. While headwater capture events are known to have occurred between the Paraíba do Sul and Upper Tietê rivers, in the Paraná basin, resulting in faunal exchanges (Buckup, 2011), no such pattern was observed here, given that the specimens from the Tietê River (*P*. aff. *australis* IX—UPA) form a distinct clade from *P*. aff. *australis* I (AML, TRO, MAD and PBS), whereas shared haplotypes were detected between the Amazonian lowlands and the Paraíba do Sul basin. While a number of endemic species are found in the Paraíba do Sul basin, the occurrence of *P. australis* is considered to be allochthonous (Sarmento‐Soares et al., 2024). The results of the present study indicate a possible Amazonian origin for these specimens, which may have been the result of the introduction of this non‐native species in this basin.

Five of the 12 lineages analysed here (IV–TOA; V–XIN; VI–XIN; VII–PAR; IX–UPA) are limited to a single basin, even though their areas of occurrence overlap with those of the other seven lineages (I–AML, TRO, MAD and PBS; II–PUR and GUA; III–PAR and GUA; VIII–PAR and UPA; X–LUR and TRA; XI–PAR, LPA, LUR and TAP; XII–PAR and TAP). Other small‐bodied fishes, such as *Characidium zebra* (Serrano et al., 2019), *Arhinolemur obtusidens* (Ramirez et al., 2017), *Bryconamericus* cf. *exodon* (García‐Melo et al., 2019), also have distinct genetic lineages distributed among two or more hydrographic basins, and many other taxa have shared lineages between the Paraguay and Paraná basins (Pereira et al., 2013).

The trans‐basin occurrence of species in the headwaters of drainage systems may result from successive orogenic headwater capture events in previously isolated basins, which can lead to the origin of new species through vicariance or enable geographic dispersal by connecting local assemblages (Albert et al., 2017, 2020). Based on the patterns observed here, two possible headwater capture events can be hypothesized—one between the Guaporé and the upper Paraguay rivers (*P*. aff. *australis* III), a faunal exchange pattern that is widely known and has been documented in other fish species (Dagosta & De Pinna, 2019; Ota et al., 2014). The other event would have involved the tributaries of the upper Tapajós and upper Paraguay rivers (*P*. aff. *australis* XI and XII), which is also known to have resulted in other cases of trans‐basin dispersal (Dagosta & De Pinna, 2019; Ribeiro et al., 2013).

Finally, the *Pyrrhulina* aff. *australis* XI lineage is widely distributed within the La Plata basin, particularly in the lowland areas that extend from the upper Paraguay River and the Pantanal to the lower Paraná River in Argentina. This continuous distribution across lowland plains sensu Lima and Ribeiro (2011), of less than 250 m above sea level, indicates that this lineage has had sustained population connectivity throughout the Paraná–Paraguay lowlands, as indicated by the high level of haplotype sharing and low genetic differentiation observed here. This pattern may be related to the annual flood pulse of the Pantanal wetlands, which will connect isolated populations periodically, a process that is especially important for species with reduced dispersal capacity (Souza et al., 2023; Quirós et al., 2007). The occurrence of specimens in the upper Tapajós River likely reflects a historical dispersal event associated with headwater capture, which reinforces the hypothesis of recurrent inter‐basin connectivity between the Tapajós and Paraguay basins (Ribeiro et al., 2013).

### 4.3 Diversification of the *P. australis* group

The MRCA of the lineages of the *P. australis* group and their western Amazonian congeners *P. obermulleri* and *P. punctata*, both from Peru, diverged in the late Miocene (5.45 Ma; 95% HPD = 2.39–8.38 Ma; Figure 5). This period coincides with intense geological events that reshaped the region's hydrographic basins, such as the end of Andean uplift in the Miocene (~10 Ma; Hubert & Renno, 2006), which generated a high tectonic load and renewed the accommodation space between adjacent basins (Hoorn et al., 2010). The simultaneous, significant increase in rainfall levels on the eastern flank of the Andes, which was intensified by tectonic processes, resulted in intensifying erosion and increased sediment and drainage input into the Amazonian lowlands (Hoorn et al., 2010). The changes in relief and the courses of rivers associated with these events likely influenced the early diversification processes within the genus *Pyrrhulina*.

The first cladogenetic event within the *P. australis* group occurred during the Pliocene, with the separation of the MRCA of *P. vittata* (MAM) from the remaining lineages of the group (4.24 Ma; 95% HPD = 2.39–8.98 Ma), which diversified more recently, forming two major clades (Figure 5), whose MRCA was dated to the Pliocene, at approximately 3.19 Ma (95% HPD = 1.49–5.44 Ma). Although the configuration of the Amazon, Paraguay and Orinoco river systems in the late Miocene to Pliocene was similar to that of the present day, the period was marked by significant fluctuations in sea level. At around 5 Ma, marine incursions raised sea levels by up to 100 m over approximately 800,000 years (~5–4.2 Ma), followed by repeated marine regressions over the past 4 million years (Hubert & Renno, 2006).

These climatic oscillations had a direct influence on population dynamics, resulting in continuous migrations and the reorganization of species in response to geological events (Webb & Bartlein, 1992). The results of the present study indicate that the diversification of the lineages of the *P. australis* group (except that of *P. vittata*) occurred primarily during the Pleistocene (2.58–0.01 Ma), a period of intense glacial cycles that reshaped lowland environments in South America. In fact, these climatic processes are widely recognized as the drivers of recent speciation in Neotropical fishes, given that they promoted the continuous expansion and contraction of habitats, resulting in the successive fragmentation and connectivity of populations (Souza et al., 2015; Mondin et al., 2018; Wendt et al., 2019).

The predominance of the study lineages in lowland river basins may reflect the phylogenetic pattern proposed by Melo et al. (2021), who suggested that these areas act as ‘macroevolutionary museums’, preserving diversity through time and eventually favouring secondary dispersal into upland basins. In part, this process may account for the ample distribution of the *P. australis* group. Even so, additional analyses, such as biogeographic modelling and tests of evolutionary scenarios, will be necessary to confirm this hypothesis more definitively.

## 5 CONCLUSIONS

The results of the present study indicate that *P. australis* comprises multiple, distinct evolutionary lineages, which are widely distributed across a number of different Neotropical river basins. The analyses indicate that the cladogenic diversification of these lineages occurred primarily during the Pleistocene. The wide geographic distribution of the specimens analysed here permitted the understanding of complex biogeographic patterns, such as possible headwater capture events between the Paraguay–Tapajós and Paraguay–Guaporé rivers, which may have further contributed to species diversification. The consistency of the results of the different analytical methods employed here reinforces the need for a thorough taxonomic review of the group, as well as a more detailed assessment of the conservation status of the different taxa, given that the true diversity of the group labelled nominally as *P. australis* has been underestimated significantly.

## AUTHOR CONTRIBUTIONS

All authors contributed to the theoretical framework and writing of the manuscript. TBS, DCF, ALNF, and PCV conceived and designed the study. TBS and CBM performed the molecular analyses. HPS and CO contributed to sample collection. TBS and DCF were responsible for editing and revising the manuscript. All authors reviewed and approved the final version of the manuscript.

## FUNDING INFORMATION

The present study was funded by *Coordenação de Aperfeiçoamento de Pessoal de Nível Superior* (CAPES, Finance Code 001 to TBS and CBM); *Fundação de Amparo à Pesquisa do Mato Grosso* (FAPEMAT‐PRO.000339/2023 to DCF); *Conselho Nacional de Desenvolvimento Científico e Tecnológico*: CNPq grant 421733/2017–9; 313834/2021‐0; 405706/2022‐7 (research productivity fellowship to ALN‐F); *Fundação de Amparo à Pesquisa do Estado do Rio Grande do Sul* (FAPERGS, grant 72550.751.48979 to ALN‐F) and the Programa Nacional de Investigación Científica y Estudios Avanzados (PROCIENCIA) within the framework of the E033‐2023‐01‐BM ‘Alianzas Interinstitucionales para Programas de Doctorado’ contest, grant number (PE501084299‐2023) to CBM.

## Supporting information


**Figure S1.** Time‐calibrated tree compiled in BEAST2 based on the age of the fossil ^†^ *Brycon avus* and the estimated mutation rate of the COI gene (see text). The different colours of the clades indicate the different families used as outgroups: Bryconidae, Chalceidae, Ctenoluciidae and Lebiasinidae. The node marked A indicates the Most Recent Common Ancestor (MRCA) of *Pyrrhulina* and *Copella* during the Oligocene (~30 Ma), while the node marked B indicates the MRCA of the *Pyrrhulina australis* group and its Amazonian congeners *P. obermulleri* and *P. punctata* in the late Miocene. The blue bars at the nodes represent the 95% Highest Posterior Density (HPD) credibility intervals for age estimates. Plio, Pliocene; Plei, Pleistocene.

**Figure S2.** Morphological variation related to the presence of the primary black stripe in specimens of *Pyrrhulina* aff. *australis* lineage I. (A) Live coloration (female) with no primary stripe; (B) male and (C) female with primary stripe; (D) male and (E) female with no primary stripe.

**Table S1.** Geographic details, sequence identification (ID), museum catalogue numbers and the BOLD Systems and GenBank accession numbers for the *Pyrrhulina* specimens included in the present study. Brazilian states: PA, Pará; AP, Amapá; RO, Rondônia; MG, Minas Gerais; MT, Mato Grosso; TO, Tocantins; SP, São Paulo; PR, Paraná; MS, Mato Grosso do Sul; SC, Santa Catarina; RS, Rio Grande do Sul.

**Table S2.** Outgroup species included in the divergence time analyses to calibrate the phylogenetic inference, together with their respective GenBank and BOLD Systems accession numbers.

## Data Availability

The data that supports the findings of this study are available in the supplementary material of this article.
