# Supplementary material for: Cryptic diversity and recent diversification in the Pyrrhulina australis species complex (Characiformes, Lebiasinidae) revealed by mitochondrial DNA
Source: J Fish Biol. 2026 Jul 2;109(3):1771–85. doi: 10.1111/jfb.70548 (PMC13602098; doi:10.1111/jfb.70548)
Supplement: Supplementary file 1 [file JFB-109-1771-s001.docx]

Table S2. Outgroup species included in the divergence time analyses to calibrate the phylogenetic inference, together with their respective GenBank and BOLD Systems accession numbers.

| **Family** | **Species** | **Number acess Genbank** | **Bold Systems** |
| --- | --- | --- | --- |
| Bryconidae | *Brycon falcatus* | - | BRY212-19 |
|  | *Brycon falcatus* | - | BRY213-19 |
|  | *Brycon falcatus* | MZ051888 | - |
|  | *Brycon insignis* | GU702086 | - |
|  | *Brycon opalinus* | GU702196 | - |
|  | *Brycon hilarii* | GU701948 | - |
|  | *Brycon orbignyanus* | GU701950 | - |
|  | *Brycon orthotaenia* | HQ600814 | - |
|  | *Brycon atrocaudatus* | OR395328 | - |
|  | *Brycon henni* | MK355252 | - |
|  | *Brycon pesu* | OR732832 | - |
|  | *Brycon nattereri* | GU701947 | - |
|  | *Brycon striatulus* | MG936820 | - |
|  | *Brycon behreae* | MG496113 | - |
|  | *Brycon chagrensis* | MG936806 | - |
|  | *Brycon guatemalensis* | MG496115 | - |
|  | *Brycon argenteus* | MG936800 | - |
|  | *Brycon obscurus* | MG936810 | - |
|  | *Brycon petrosus* | MG936814 | - |
|  | *Brycon rubricauda* | PQ233684 | - |
|  | *Brycon oligolepis* | PQ233773 | - |
|  | *Brycon fowleri* | PQ233443 | - |
|  | *Brycon moorei* | PQ233693 | - |
|  | *Brycon melanopterus* | OM453697 | - |
|  | *Salminus brasiliensis* | KY441734 | - |
|  | *Salminus hilarii* | KY441747 | - |
|  | *Henochilus wheatlandii* | - | BRY358-19 |
|  | *Henochilus wheatlandii* | - | BRY359-19 |
| Chalceidae | *Chalceus macrolepidotus* | MZ051902 | - |
|  | *Chalceus macrolepidotus* | MZ052030 | - |
| Ctenoluciidae | *Boulengerella maculata* | - | AMZON044-24 |
|  | *Boulengerella maculata* | - | AMZON043-24 |
|  | *Boulengerella cuvieri* | OR732804 | - |
|  | *Boulengerella cuvieri* | OR732805 | - |
|  | *Ctenolucius hujeta* | JF800932 | - |
|  | *Ctenolucius beani* | MG936853 | - |
| Lebiasinidae | *Lebiasina boruca* | MG496168 | - |
|  | *Lebiasina bimaculata* | OR395344 | - |
|  | *Nannostomus eques* | OR733052 | - |
|  | *Nannostomus eques* | OR733050 | - |
|  | *Nannostomus eques* | PX879049 (present estudy) | - |
|  | *Copella arnoldi* | GBOL2183 | - |
|  | *Copella arnoldi* | GBOL2184 | - |
|  | *Copella calloleps* | PX879046 (present estudy) | - |
|  | *Copella* sp. | PX879047 (present estudy) | - |
|  | *Copella* sp. | PX879048 (present estudy) | - |
