# Supplementary material for: Cryptic diversity and recent diversification in the Pyrrhulina australis species complex (Characiformes, Lebiasinidae) revealed by mitochondrial DNA
Source: J Fish Biol. 2026 Jul 2;109(3):1771–85. doi: 10.1111/jfb.70548 (PMC13602098; doi:10.1111/jfb.70548)
Supplement: Supplementary file 2 [file JFB-109-1771-s004.docx]

**Table S1.** Table S1. Geographic details, sequence identification (ID), museum catalog numbers, and the BOLD Systems and GenBank accession numbers for the *Pyrrhulina* specimens included in the present study. Brazilian states: PA = Pará, AP = Amapá, RO = Rondônia, MG = Minas Gerais, MT = Mato Grosso, TO = Tocantins, SP = São Paulo, PR = Paraná, MS = Mato Grosso do Sul, SC = Santa Catarina, and RS = Rio Grande do Sul.

| **MOTU/Lineage** | **Locality** | **River/Drainage Basin** | **Latitude** | **Longitude** | **ID sequence** | **Voucher Museum** | **Number access BOLD/ Genbank** |
| --- | --- | --- | --- | --- | --- | --- | --- |
| *P.* aff. *australis* I- Amazon Lowland, Trombetas, Madeira and Paraíba do Sul | Óbidos, PA, Brazil | Cuminá/Trombetas/Amazon | -1.171211 | -55.68695 | 115414 | LBP 34270 | PX878928 |
| *P.* aff. *australis* I- Amazon Lowland, Trombetas, Madeira and Paraíba do Sul | Calçoene, AP, Brazil | Calçoene/Amazon | 2.479917 | -50.97977 | 81271 | LBP 21030 | PX878929 |
| *P.* aff. *australis* I- Amazon Lowland, Trombetas, Madeira and Paraíba do Sul | Calçoene, AP, Brazil | Calçoene/Amazon | 2.519138 | -51.01469 | 81980 | LBP 21015 | PX878930 |
|  |  |  |  |  | 81981 |  | PX878931 |
| *P.* aff. *australis* I- Amazon Lowland, Trombetas, Madeira and Paraíba do Sul  *P.* aff. *australis* I- Amazon Lowland, Trombetas, Madeira and Paraíba do Sul | Juruti, PA, Brazil | Amazonas/Amazon | -2.31006 | -55.78254 | 11347 | CPUFMT 8770 | PX878932 |
|  |  |  |  |  | 11350 |  | PX878933 |
| *P.* aff. *australis* I- Amazon Lowland, Trombetas, Madeira and Paraíba do Sul  *P.* aff. *australis* I- Amazon Lowland, Trombetas, Madeira and Paraíba do Sul  *P.* aff. *australis* I- Amazon Lowland, Trombetas, Madeira and Paraíba do Sul | Comunidade Curuai, Satarém, PA, Brazil | Amazonas/Amazon | -2.245291 | -55.493414 | 11398 | CPUFMT 8771 | PX878934 |
|  |  |  |  |  | 11403 |  | PX878935 |
|  |  |  |  |  | 11406 |  | PX878936 |
| *P.* aff. *australis* I- Amazon Lowland, Trombetas, Madeira and Paraíba do Sul | Guajará Mirim, RO, Brazil | Madeira/Amazon | -10.23177 | -65.283 | 50633 | LBP 11043 | PX878937 |
| *P.* aff. *australis* I- Amazon Lowland, Trombetas, Madeira and Paraíba do Sul | Angaturana, MG, Brazil | Paraíba do Sul/ Southeast Atlantic | -21.4411 | -42.438722 | 20341 | LBP 3430 | PX878938 |
| *P.* aff. *australis* II- Purus and Guaporé | Beruri, Amazonas, Brazil | Purus/Amazon | -4.274722 | -61.87872 | 103331 | LBP 29511 | PX878939 |
| *P.* aff. *australis* II- Purus and Guaporé | Vila Bela da Santíssima Trindade, MT, Brazil | Guaporé/ Amazon | -14.969027 | -59.983194 | 49926 | LBP 10801 | PX878940 |
| *P*. aff. *australis* III- Paraguay and Guaporé | Pontes e Lacerda, MT, Brazil | Guaporé/Amazon | -15.3584 | -59.252934 | 11111 | CPUFMT 7762 | PYR001-22 |
|  |  |  |  |  | 11112 |  | PYR002-22 |
|  |  |  |  |  | 11113 |  | PYR003-22 |
|  |  |  |  |  | 11114 |  | PYR004-22 |
|  |  |  |  |  | 11115 |  | PYR005-22 |
| *P*. aff. *australis* III- Paraguay and Guaporé | Vila Bela da Santíssima Trindade, MT, Brazil | Guaporé/Amazon | -14.969027 | -59.983194 | 49929 | LBP 10801 | PX878941 |
| *P*. aff. *australis* III- Paraguay and Guaporé | Comodoro, MT, Brazil | Guaporé/Amazon | -14.057222 | -59.590833 | 11638 | CPUFMT 8773 | PX878942 |
|  |  |  |  |  | 11639 |  | PX878943 |
| *P*. aff. *australis* III- Paraguay and Guaporé | Reserva do Cabaçal, MT, Brazil | Upper Paraguay | -15.123387 | -58.395359 | 11446 | CPUFMT 8774 | PX878944 |
|  |  |  |  |  | 11447 |  | PX878945 |
|  |  |  |  |  | 11448 |  | PX878946 |
|  |  |  |  |  | 11449 |  | PX878947 |
| *P.* aff. *australis* IV- Tocantins- Araguaia | Porto Alegre do Norte, MT, Brazil | Araguaia | -11.261667 | -51.605 | 1924B | UFRGS 15471 | PX878948 |
|  |  |  |  |  | 1924C |  | PX878949 |
| P. aff. australis IV- Tocantins- Araguaia | Barra do Garças, MT, Brazil | Rio das Mortes/Araguaia | -15.682694 | -52.22355 | 16285 | LBP 2431 | PX878950 |
|  |  |  |  |  | 16286 |  | PX878951 |
| P. aff. australis IV- Tocantins- Araguaia | Brejinho de Nazaré, Tocantins, Brazil | Crixá /Upper Tocantins | -11.053972 | -48.5727 | 77564 | LBP 19192 | PX878952 |
|  |  |  |  |  | 77565 |  | PX878953 |
| *P.* aff. *australis* IV- Tocantins- Araguaia | Barra do Garças, MT, Brazil | Rio das Garças /Upper Araguaia | -15.778658 | -52.2768 | 23 | CPUFMT 7758 | PYR026-22 |
|  |  |  |  |  | 24 |  | PYR027-22 |
|  |  |  |  |  | 25 |  | PYR028-22 |
|  |  |  |  |  | 26 |  | PYR029-22 |
|  |  |  |  |  | 27 |  | PYR030-22 |
| *P.* aff. *australis* V- Xingu | Querência, MT, Brazil | Feio/Xingu | -12.555694 | -52.271138 | 64832 | LBP 15803 | PX878954 |
|  |  |  |  |  | 64833 | LBP 15803 | PX878955 |
| *P.* aff. *australis* VI- Xingu | Serrinha, Água Boa, MT, Brazil | Coluene/Xingu | -13.730194 | -53.0322 | 66271 | LBP 16029 | PX878956 |
|  |  |  |  |  | 66272 | LBP 16029 | PX878957 |
| *P.* aff. *australis* VII- Paraguay | Itiquira, MT, Brazil | Correntes/ Paraguay | -17.53447 | -54.42683 | 11420 | LABGEN | PX878958 |
|  |  |  |  |  | 11421 | LABGEN | PX878959 |
|  |  |  |  |  | 11423 | LABGEN | PX878960 |
| *P.* aff. *australis* VIII- Paraguay and Upper Paraná | Uberaba, MG, Brazil | Grande/ Upper Paraná | -20.054167 | -47.878333 | 1846B | UFRGS 15062 | PX878961 |
| *P.* aff. *australis* VIII- Paraguay and Upper Paraná | Itiquira, MT, Brazil | Piquiri/ Upper Paraguay | -17.470277 | -55.246306 | 14079 | LBP 1935 | PX878962 |
| *P.* aff. australis VIII- Paraguay and Upper Paraná | Aquidauana, MS, Brazil | Negro/ Paraguay | -19.571472 | -56.24577 | 223333 | LBP 3784 | PX878963 |
| P. aff. *australis* VIII- Paraguay and Upper Paraná | Coxim, MS, Brazil | Taquari/ Paraguay | -18.422722 | -54.835083 | 14050 | LBP 1921 | PX878964 |
|  |  |  |  |  | 14051 |  | PX878965 |
| *P.* aff. *australis* VIII- Paraguay and Upper Paraná | Poconé, Cangas- MT, Brazil | Bento Gomes/ Upper Paraguay | -16.152052 | -56.617459 | 10995 | CPUFMT 7757 | PYR035-22 |
| *P.* aff. *australis* VIII- Paraguay and Upper Paraná | Corumbá, MS, Brazil | Miranda/ Paraguay | -19.009619 | -57.000312 | 3634 | LBP 47 | PX878966 |
| *P.* aff. *australis* VIII- Paraguay and Upper Paraná | Formosa, Argentina | Paraguay/ Lower Paraguay | -26.1375 | -58.124769 | FWFA1052-15 | BOLD Systems | FWFA1052-15 |
| *P.* aff. *australis* VIII- Paraguay and Upper Paraná | Cravinhos, SP, Brazil | Pardo/ Upper Paraná | -21.3775 | -47.6592 | FUPR018-09 | BOLD Systems | FUPR018-09 |
|  |  |  |  |  | FUPR019-09 |  | FUPR019-09 |
| *P.* aff. *australis* IX- Upper Paraná | Porto Rico, PR, Brazil | Paraná-Oeste/ Upper Paraná | -22.71755 | -53.291 | 17352 | LBP 2632 | PX878967 |
| *P.* aff. *australis* IX- Upper Paraná | Diamante do Norte, PR, Brazil | Paranapanema/ Upper Paraná | -22.634722 | -52.82211 | 44412 | LBP 8932 | PX878968 |
|  |  |  |  |  | 44413 |  | PX878969 |
| *P.* aff. *australi*s IX- Upper Paraná | Naviraí, MS, Brazil | Laranjal/ Upper Paraná | -23.138639 | -53.806167 | 11972 | CPUFMT 8793 | PX878970 |
|  |  |  |  |  | 11982 |  | PX878971 |
| *P.* aff. *australis* IX- Upper Paraná | Angelica, MS, Brazil | Paraná/ Upper Paraná | -22.13325 | -53.76911 | 45612 | LBP 9631 | PX878972 |
|  |  |  |  |  | 45613 |  | PX878973 |
| *P.* aff. *australis* IX- Upper Paraná | Botucatu, SP, Brazil | Tietê/ Upper Paraná | -22.7555 | -48.30685 | 47656 | LBP 8740 | PX878974 |
|  |  |  |  |  | 47657 |  | PX878975 |
| *P.* aff. *australis* IX- Upper Paraná | Poloni, SP, Brazil | Tietê/ Upper Paraná | -20.745472 | -49.77925 | 17391 | LBP 2607 | PX878976 |
| *P.* aff. *australis* IX- Upper Paraná | Marilena, PR, Brazil | Paranapanema/ Upper Paraná | -22.6734 | -53.0858 | FUPR020-09 | BOLD Systems | FUPR020-09 |
|  |  |  |  |  | FUPR021-09 | BOLD Systems | FUPR021-09 |
|  |  |  |  |  | FUPR022-09 | BOLD Systems | FUPR022-09 |
| *P.* aff. *australis* IX- Upper Paraná | Nova Andradina, MS, Brazil | Ivinhema/ Upper Paraná | -22.039138 | -53.52316 | 45752 | LBP 9677 | PX878977 |
|  |  |  |  |  | 45753 |  | PX878978 |
| *P.* aff. *australis* X- Lower Uruguay and Tramandaí | Sombrio, SC, Brazil | Tramandaí/ South Atlantic | -29.042972 | -49.576222 | 2929 | UFRGS 16601 | PX878979 |
| *P.* aff. *australis* X- Lower Uruguay and Tramandaí | Manoel Viana, RS, Brazil | Uruguay | -29.201333 | -55.496028 | 6430E | UFRGS 21416 | PX878980 |
|  |  |  |  |  | 6430D |  | PX878981 |
| *P.* aff. *australis* X- Lower Uruguay and Tramandaí | Torres, RS, Brazil | Tramandaí/ South Atlantic | -29.409781 | -49.799033 | 4873A | UFRGS 19155 | PX878982 |
|  |  |  |  |  | 4873C |  | PX878983 |
|  |  |  |  |  | 4873F |  | PX878984 |
| *P.* aff. *australi*s + XI- Paraguay, Lower Paraná, Lower Uruguay and Tapajós | Uruguaiana, RS, Brazil | Lower Uruguay | -29.5118 | -56.719416 | 55118 | LBP 13173 | PX878985 |
|  |  |  |  |  | 55119 |  | PX878986 |
| *P.* aff. *australis* XI- Paraguay, Lower Paraná, Lower Uruguay and Tapajós | Uruguaiana, RS, Brazil | Lower Uruguay | -29.497917 | -56.595361 | 7664 | UFRGS 23635 | PX878987 |
| *P.* aff. *australis* XI- Paraguay, Lower Paraná, Lower Uruguay and Tapajós | Uruguaiana, RS, Brazil | Lower Uruguay | -29.884083 | -57.11575 | 7539B | UFRGS 23445 | PX878988 |
| *P.* aff. *australis* XI- Paraguay, Lower Paraná, Lower Uruguay and Tapajós | Formosa, Rdeg Tohue, Argentina | Lower Paraguay | -26.1375 | -58.124769 | FWFA445-15 | BOLD Systems | FWFA445-15 |
|  |  |  |  |  | FWFA446-15 |  | FWFA446-15 |
|  |  |  |  |  | FWFA493-15 |  | FWFA493-15 |
| *P.* aff. *australis* XI- Paraguay, Lower Paraná, Lower Uruguay and Tapajós | Corrientes, Argentina | Lower Paraná | -28.219 | -58.806 | MLPEC317-12 | BOLD Systems | MLPEC317-12 |
|  |  |  |  |  | MLPEC318-12 |  | MLPEC318-12 |
|  |  |  |  |  | MLPEC319-12 |  | MLPEC319-12 |
| *P.* aff. *australis* XI- Paraguay, Lower Paraná, Lower Uruguay and Tapajós | Victoria, Argentina | Lower Paraná | -32.887 | -60.669 | LARI315-13 | BOLD Systems | LARI315-13 |
|  |  |  |  |  | LARI316-13 |  | LARI316-13 |
| *P.* aff. *australis* XI- Paraguay, Lower Paraná, Lower Uruguay and Tapajós | Cuiabá, Coxipó da Ponte, MT, Brazil | Aricá-açu/ Upper Paraguay | -15.6194444 | -55.700083 | 10790 | CPUFMT 8778 | PX878989 |
|  |  |  |  |  | 10791 |  | PX878990 |
| *P.* aff. *australis* XI- Paraguay, Lower Paraná, Lower Uruguay and Tapajós | Cuiabá, Coxipó da Ponte, MT, Brazil | Aricá-açu/ Upper Paraguay | -15.5596388 | -55.789972 | 10794 | CPUFMT 8776 | PX878991 |
|  |  |  |  |  | 10796 |  | PX878992 |
| *P.* aff. *australis* XI- Paraguay, Lower Paraná, Lower Uruguay and Tapajós | Cuiabá, Coxipó da Ponte, MT, Brazil | Aricá-açu/ Upper Paraguay | -15.5368333 | -55.892027 | 10803 | CPUFMT 8775 | PX878993 |
| *P.* aff. *australis* XI- Paraguay, Lower Paraná, Lower Uruguay and Tapajós | Mimoso, Santo Antônio do Leverger, MT, Brazil | Cuiabá/ Upper Paraguay | -16.318818 | -55.822598 | 7084 | CPUFMT 8779 | PX878994 |
|  |  |  |  |  | 7085 |  | PX878995 |
|  |  |  |  |  | 7086 |  | PX878996 |
| *P.* aff. *australis* XI- Paraguay, Lower Paraná, Lower Uruguay and Tapajós | Santo Antônio do Leverger, MT, Brazil | Cuiabá/ Upper Paraguay | -15.749291 | -56.081296 | 11575 | CPUFMT 8780 | PX878997 |
|  |  |  |  |  | 11576 |  | PX878998 |
|  |  |  |  |  | 11577 |  | PX878999 |
|  |  |  |  |  | 11578 |  | PX879000 |
|  |  |  |  |  | 11579 |  | PX879001 |
| *P.* aff. *australis* XI- Paraguay, Lower Paraná, Lower Uruguay and Tapajós | Santo Antônio do Leverger, MT, Brazil | Aricá-Mirim/ Upper Paraguay | -15.49283333 | -55.971222 | 10850 | CPUFMT 8781 | PX879002 |
|  |  |  |  |  | 10851 |  | PX879003 |
| *P.* aff. *australis* XI- Paraguay, Lower Paraná, Lower Uruguay and Tapajós | Santo Antônio do Leverger, MT, Brazil | Coxipó/ Upper Paraguay | -15.76008333 | -55.62775 | 10652 | CPUFMT 8782 | PX879004 |
|  |  |  |  |  | 10871 |  | PX879005 |
|  |  |  |  |  | 10874 |  | PX879006 |
|  |  |  |  |  | 10875 |  | PX879007 |
|  |  |  |  |  | 10877 |  | PX879008 |
|  |  |  |  |  | 10878 |  | PX879009 |
| *P.* aff. *australis* XI- Paraguay, Lower Paraná, Lower Uruguay and Tapajós | Corumbá, MS, Brazil | São Lourenço/ Paraguay | -17.840839 | -57.397372 | 10890 | CPUFMT 8784 | PX879010 |
|  |  |  |  |  | 10891 |  | PX879011 |
|  |  |  |  |  | 10901 |  | PX879012 |
|  |  |  |  |  | 10902 |  | PX879013 |
|  |  |  |  |  | 10903 |  | PX879014 |
|  |  |  |  |  | 10904 |  | PX879015 |
| *P.* aff. *australis* XI- Paraguay, Lower Paraná, Lower Uruguay and Tapajós | Cáceres, MT, Brazil | Jauru/ Upper Paraguay | -16.344604 | -57.791577 | 10897 | CPUFMT 8785 | PX879016 |
|  |  |  |  |  | 10898 |  | PX879018 |
|  |  |  |  |  | 10899 |  | PX879017 |
|  |  |  |  |  | 10900 |  | PX879019 |
| *P.* aff. *australis* XI- Paraguay, Lower Paraná, Lower Uruguay and Tapajós | Poconé, MT, Brazil | Piraim/ Upper Paraguay | -16.364707 | -56.292097 | 10938 | CPUFMT 8786 | PX879020 |
|  |  |  |  |  | 10939 |  | PX879021 |
|  |  |  |  |  | 10940 |  | PX879022 |
|  |  |  |  |  | 10943 |  | PX879023 |
| *P.* aff. *australis* XI- Paraguay, Lower Paraná, Lower Uruguay and Tapajós | Pirizal, Nossa do Livramento, MT, Brazil | Santana/ Upper Paraguay | -16.231707 | -56.392414 | 10961 | CPUFMT 8787 | PX879024 |
|  |  |  |  |  | 10962 |  | PX879025 |
|  |  |  |  |  | 10964 |  | PX879026 |
| *P.* aff. *australis* XI- Paraguay, Lower Paraná, Lower Uruguay and Tapajós | Poconé, Cangas, MT, Brazil | Bento Gomes/ Upper Paraguay | -16.152052 | -56.617459 | 10991 | CPUFMT 7757 | PYR031-22 |
|  |  |  |  |  | 10992 |  | PYR032-22 |
|  |  |  |  |  | 10993 |  | PYR033-22 |
|  |  |  |  |  | 10994 |  | PYR034-22 |
| *P.* aff. *australis* XI- Paraguay, Lower Paraná, Lower Uruguay and Tapajós | Barra do Bugres, MT, Brazil | Upper Paraguay | -15.15408 | -56.95498 | 11442 | CPUFMT 8788 | PX879027 |
|  |  |  |  |  | 11444 |  | PX879028 |
|  |  |  |  |  | 11445 |  | PX879029 |
| *P.* aff. *australis* XI- Paraguay, Lower Paraná, Lower Uruguay and Tapajós | Araputanga, MT, Brazil | Upper Paraguay | -15.46577 | -58.2996 | 11429 | CPUFMT 8795 | PX879030 |
| *P.* aff. *australis* XI- Paraguay, Lower Paraná, Lower Uruguay and Tapajós | Utiariti, Campo Novo do Parecis, MT, Brazil | Sacre/ Tapajós/ Amazon | -13.2375 | -58.131944 | 11993 | CPUFMT 8789 | PX879031 |
|  |  |  |  |  | 11994 |  | PX879032 |
| *P.* aff. *australis* XII- Paraguay and Tapajós | Tangará da Serra, MT, Brazil | Sepotuba/ Upper Paraguay | -14.34967 | -57.53046 | 11430 | CPUFMT 8790 | PX879033 |
|  |  |  |  |  | 11431 |  | PX879034 |
|  |  |  |  |  | 11432 |  | PX879035 |
|  |  |  |  |  | 11433 |  | PX879036 |
| *P.* aff. *australi*s XII- Paraguay and Tapajós | Tangará da Serra, MT, Brazil | Sepotuba/ Upper Paraguay | -14.35055 | -57.6346 | 41428 | LBP 9037 | PX879037 |
|  |  |  |  |  | 41429 |  | PX879038 |
| P. aff. australis XII- Paraguay and Tapajós | Deciolância, Diamantino, MT, Brazil | Tapajós/ Amazon | -14.175931 | -57.515486 | 11338 | CPUFMT 8791 | PX879039 |
| P. aff. australis XII- Paraguay and Tapajós | Diamantino, MT, Brazil | Arinos/ Tapajós/ Amazon | -13.899225 | -57.084801 | 81521 | LBP 20884 | PX879040 |
|  |  |  |  |  | 81522 |  | PX879041 |
| *P. marilynae-* Tapajós | Sorriso, MT, Brazil | Teles Pires/ Tapajós/ Amazon | -12.90307 | -55.71256 | 11434 | CPUFMT 8792 | PX879042 |
| *P. marilynae-* Tapajós | Sorriso, MT, Brazil | Teles Pires/ Tapajós/ Amazon | -12.95491 | -55.74384 | 11093 | CPUFMT 7759 | PYR023-22 |
| *P. vittata-* Mamoré | Guajara-Mirim, RO, Brazil | Mamoré/ Madeira/Amazon | -11.571388 | -65.1425 | 13115 | UFRO-ICT 027714 | PX879043 |
| *P. obermulleri*- Amazon Lowland | Nauta, Loreto, Peru | Marañon/ Amazon | -4.01283 | -73.46325 | 54231 | LBP 12570 | PX879044 |
|  |  |  |  |  | 54232 |  | PX879045 |
| *P. punctata-* Amazon Lowland | Quebrada Monterriguillo, Loreto, Peru | Tigre/ Amazon | -3.185444 | -75.027167 | LOR1 | LBP 31762 | PV719272 |
|  |  |  |  |  | LOR2 |  | PV719273 |
